# Enhancing Teaching Recovery Techniques (TRT) with Parenting Skills: RCT of TRT + Parenting with Trauma-Affected Syrian Refugees in Lebanon Utilising Remote Training with Implications for Insecure Contexts and COVID-19 †

**DOI:** 10.3390/ijerph18168652

**Published:** 2021-08-16

**Authors:** Aala El-Khani, Kim Cartwright, Wadih Maalouf, Karin Haar, Nosheen Zehra, Gökçe Çokamay-Yılmaz, Rachel Calam

**Affiliations:** 1 Prevention, Treatment and Rehabilitation Section, Drug Prevention and Health Branch, Division of Operations, United Nations Office on Drugs and Crime (UNODC), Wagramer Strasse 5, A-1400 Vienna, Austria; Wadih.maalouf@un.org (W.M.); Karin.haar@un.org (K.H.); 2 Division of Psychology & Mental Health, University of Manchester, Manchester M13 9WL, UK; Rachel.calam@manchester.ac.uk; 3 Greater Manchester Mental Health NHS Foundation Trust, Complex Trauma and Resilience Research Unit, Research and Innovation Office, Manchester M13 9WL, UK; Kim.Cartwright@gmmh.nhs.uk; 4 Global Mental Health and Cultural Psychiatry Research Group, Manchester M13 9PL, UK; Nosheen_zehra130@hotmail.com; 5 Greater Manchester Mental Health NHS Foundation Trust, Manchester M23 9LT, UK; Gokce.Cokamay@gmmh.nhs.uk

**Keywords:** refugee, family skills, parenting, displaced population, war, conflict

## Abstract

Child psychosocial recovery interventions in humanitarian contexts often overlook the significant effect that caregivers can have on improving children’s future trajectory. We enhanced the well-established, evidenced-based child trauma recovery programme Teaching Recovery Techniques (TRT) intervention with parenting sessions, i.e., TRT + Parenting (TRT + P), which aims to improve parent mental health and their ability to support their children’s mental health. We describe the findings of a three-arm randomised controlled trial comparing enhanced TRT + P vs. TRT and waitlist. The primary aim was to test if children in the enhanced arm of the programme show improved child and caregiver mental health. We recruited 119 Syrian refugee children and one of their caregivers in Beqaa Valley in Lebanon. They were randomised to the TRT, TRT + P, or waitlist control group. Data were collected at baseline and 2 weeks and 12 weeks post intervention. Training of facilitators was via remote training from the United Kingdom. Results showed a highly consistent pattern, with children in the enhanced TRT + P group showing the greatest levels of improvement in behavioural and emotional difficulties compared to children in the TRT or waitlist control groups. Caregivers in the TRT + P group also reported significant reductions in depression, anxiety, and stress. Findings indicate that the addition of the evidence-based parenting skills components has the potential to enhance the effects of interventions designed to improve children’s mental health in contexts of trauma, conflict, and displacement. Implications for COVID-19 remote learning are also discussed.

## 1. Introduction

We are living through one of the greatest humanitarian crises of our time. Current global conflicts have contributed to the highest rates of displacement on record, with an estimated 79.5 million people forcibly displaced [1]. An estimated one in five children in the world now lives in areas affected by armed conflict [2].

Epidemiological research in populations that have experienced conflict and displacement indicates much higher levels of psychopathology, mainly post-traumatic stress disorder (PTSD), depression, anxiety, and behavioural problems [3,4]. Exposure to armed conflict has detrimental effects on children’s mental health, development, and life opportunities [5]. These effects create a burden on the countries into which children are displaced [6]. Children living in conflict-affected settings often face cognitive, physical, and social–emotional challenges that affect how they learn, grow, and interact with others. There is evidence that some forms of psychosocial intervention can have significant benefits for these children [7]. Families have a key role to play, yet research is limited on how to best support war-affected caregivers in caring for their children [8].

A supportive environment with nurturing caregivers is essential for the healthy development of children [9,10], and this has been established in diverse cultural and social contexts [11]. For children who have been exposed to conflict and displacement, the need for strong healthy, nurturing caregiver relationships may assume even greater importance [12] when other extended support systems in their lives, such as community and extended family, have been destroyed. Positive family interactions can serve as a protective factor as families face the upheaval of migration [13,14]. Studies which explored the linkages between war and parenting in ongoing humanitarian crisis highlighted the key role that parents or primary caregivers play [8]. For example, in a study of 710 Lebanese adolescents who had recently been exposed to war, resilience was associated with having parents who spent time with them and supported them [15].

Conversely, research provides clear evidence that certain family characteristics can act as strong risk factors which are associated with a variety of youth problem behaviour [16,17]. Thus, an insufficient family support system can increase the chances of children developing behaviour problems. Longitudinal studies in Afghanistan, Pakistan, and Sierra Leone have demonstrated the enduring impact of war and how family variables, specifically including the care provided by primary caregivers, can continue to affect the next generation, predicting mental health outcomes in children, over and above actual traumatic experiences [18].

Family skills interventions have been found to be effective in encouraging safe and nurturing relationships between parents (or caregivers) and children in their early years and, as such, preventing many problem behaviours including violence [19,20]. There is much evidence of the effectiveness of parenting and family skills interventions in high-income and stable contexts in improving caregiver–child communication and relationships, parenting skills, and the mental health of family members. However, few interventions have been tested in low- and middle-income countries, along with even fewer in contexts of conflict and displacement [21,22]. In light of increasing awareness in recent years of the significant role that parents play, family-based approaches including both caregivers and their children in psychosocial care are becoming more recognised as a key approach in supporting families that have experienced conflict and displacement [23,24,25].

Given the growth of unprecedented numbers of people living in conflict zones or displaced globally as refugees, and in order to advance human development globally, consistent with UN Sustainable Development Goals, it is more important than ever that effective interventions are developed and implemented to promote the wellbeing of all family members, prevent further emotional and behaviour challenges, and respond to current concerns.

### The Current Study

Syrians represent the largest refugee population in the world, with over 5.6 million having left Syria since the conflict began in 2011, with an equal number internally displaced [26]. There has been a surge in research conducted to address the gap in empirical evidence on how the crisis is affecting children and families, with ecological models being developed to describe important processes and variables in transition and associated risks and protective factors [27,28]. Syrian refugees have been exposed to multiple war-related stressors before arriving into their host countries, including torture and witnessing the death of family members and destruction of their homes and livelihood [29]. A wide range of mental health problems exist, including exacerbations of pre-existing mental disorders, as well as new problems caused by the traumatic experiences of the conflict and the multiple losses they have endured [30]. Research with displaced Syrian caregivers has found high levels of empathy for the challenges that their children face, yet psychological distress has been found to contribute to harsh and violent parenting [8,31]. Caregivers in refugee camps on the Syrian–Turkish border [31], in the Za’atari refugee camp in Jordan, and in a conflict zone in northern Syria [32] reported urgently wanting help in understanding how best to parent their children, but having little or no access to such support with families. Humanitarian workers also described parenting support as a pressing need.

Brief, resource-light, protective mental health and psychosocial interventions which can be delivered by non-specialists with minimal training, and that can be incorporated into humanitarian and development responses to allow wide and rapid dissemination are urgently needed. One example of a programme which has been designed for trauma affected populations in humanitarian settings is the Children and War Foundation’s evidence-based Teaching Recovery Techniques (TRT) programme [33]. This programme is based on trauma-focused cognitive behavioural therapy for children. TRT was explicitly designed to meet the needs of low-resource settings where large numbers of children are in need, and it is aimed at reducing post-traumatic stress (PTS) in children and adolescents exposed to conflict. TRT was developed from the findings of numerous studies across significant humanitarian contexts, as well as war-affected countries hosting refugee children, with positive effects reported. High acceptability of TRT has been reported together with large effect sizes in reducing signs of PTSD, depression, and grief in the context of conflict in Palestine [34]. Moderate effects have been found in Gaza [35]. A study of unaccompanied refugee minors conducted in Sweden showed a significant decrease in PTSD and depression [36]. TRT training for those working with Syrian refugees has taken place in Lebanon and Turkey [37], but no systematic data are available yet for families displaced by the Syrian conflict. Reports on Mental Health and Psychosocial Support (MHPSS) provision for displaced Syrians recommended that programmes such as TRT, which have been validated elsewhere, are promising but require evaluation in the Syrian context [38].

The present study, conducted in Lebanon, tested the effects of combining parenting skills intervention with TRT. An enhanced version of TRT, TRT + Parenting (TRT + P), was developed. The additional parenting components were designed in response to needs identified by displaced Syrian families [23,31]. They comprised additional caregiver skills sessions designed to help build self-regulatory skills and reduce child emotional and behavioural difficulties, alongside the original TRT intervention. A pilot study of TRT + P with Syrian refugees in Turkey indicated positive feasibility and that the programme could have the potential to reduce child post-traumatic stress while also increasing parental efficacy and effective caregiving strategies [23].

Since 2009, the United Nations Office on Drugs and Crime (UNODC) has been promoting evidence-based prevention in low- and middle-income countries (LMICs). UNODC has focused on piloting evidence-based or evidence-informed preventive interventions adapted to national needs and documenting evaluation reports on their process of implementation, effectiveness, and cost-effectiveness. Family-based prevention programmes are seen as a key component. Over 23 countries in more than five geographical locations have been engaged in adapting and piloting family skills initiatives [39]. The UNODC office in Lebanon is part of this global initiative and has been actively engaged with local governmental counterparts on capacity-building programming. This office facilitated the implementation of this trial.

The aim of the current study was to test the effectiveness of TRT + P in enhancing both short- and longer-term outcomes for children and caregivers displaced by the Syrian conflict and residing in Beqaa in Lebanon. It compared the new TRT + P enhanced programme to the original TRT and to a waitlist condition. In particular, it tested the levels of reduction in children’s trauma related stress and mental health difficulties and the enhancement of parental mental health and positive parenting skills and confidence. It was predicted that outcomes would be enhanced with the addition of the parenting skills component.

## 2. Materials and Methods

### 2.1. Study Design

This study was a three-arm RCT with participants randomly allocated to the TRT + P group (intervention), a TRT group (active comparison), or a waitlist control group. Outcome measures were completed by participants at baseline (1 week before intervention delivery: T1) and 2 weeks (T2) and 12 weeks (T3) after intervention delivery. Three measures were completed by participating children and five were completed by participating adult caregivers. A demographic and background experience questionnaire was also completed at T1 by caregivers.

### 2.2. Study Setting

The study was conducted in a village in the Beqaa Valley in Lebanon close to the Western Syrian border. There are an estimated 880,000 refugees living across Lebanon, with almost half of these refugees living in the Beqaa region [26]. Participants were recruited via self-referral from three schools that the Lebanese NGO Social Support Society (SSS) provides services for. SSS operates educational and psychosocial programmes for Syrian refugee children and their caregivers in Lebanon.

### 2.3. Ethics and Governance

The study received ethical approval from the University of Manchester Research Ethics Committee. It was also reviewed and approved by the UNODC Drug Prevention and Health Branch in both the headquarters office in Vienna and the national field office in Beirut, Lebanon. This study was performed in accordance with the ethical standards of the 1964 Helsinki Declaration and its later amendments or comparable ethical standards.

### 2.4. The Intervention 

Teaching Recovery Techniques (TRT) [33] is an evidence-based, manualised intervention with a clear protocol and step-by-step practical workbook. It aids professionals working with children aged 8 and older in conflict and displacement settings in teaching skills and techniques which are helpful in coping with the psychological effects of war and violence. It is a preventive programme, intended to reduce the need for later treatment. TRT comprises five 2 h child sessions delivered in groups of up to 15 children which cover memories, nightmares, flashbacks, difficulties in relaxing, concentrating, and sleeping, and fears associated with reminders of war. In addition, TRT includes two parallel 2 h parent/caregiver sessions which cover normalising children’s reactions and helping caregivers to help themselves.

TRT’s two original parent sessions do not provide detail on caregiver needs and challenges experienced through conflict and displacement, as well as the caregiver needs identified by recent studies in understanding emotional and behavioural change in children, appropriate discipline techniques, and ways of building better communication with affected children. Three additional caregiver TRT sessions were, therefore, developed. These new sessions were designed to complement TRT’s two parent sessions and included evidence-based parenting strategies to build self-regulatory skills and reduce child emotional and behavioural difficulties. These generic, active ingredients in parent skills programmes were identified via systematic, meta-analytic, and combinative approaches [16,40]. These skills were combined with identified needs, areas for focus, and examples drawn from interviews with parents and caregivers living through conflict and displacement and the literature from a wide range of sources on the refugee experience [31].

The new parenting skills components (+ Parenting) included understanding behavioural changes, increasing positive parent–child interactions through taking time to notice and praise desirable behaviour, effective, consistent disciplinary approaches with the use of simple techniques such as behaviour charts to reward desirable behaviour, using materials that might be available in a low-resource environment, techniques for using ignoring or quiet time to reduce unwanted behaviours, staying calm, how to establish routines even in difficult contexts, listening and communicating effectively, encouraging play, making use of available resources and attending to safety, caregivers taking care of themselves, helping caregivers to plan for the future, including security concerns, and building communities in settings of displacement. This enhanced version of the TRT programme, TRT + P, was used in this study. The programme was designed for any primary caregiver having responsibility for a child. The term “parenting” is used in relation to evidence-derived parenting skills.

### 2.5. Participants and Eligibility Criteria

Participants were families with children who had been displaced by the Syrian conflict and were living in Beqaa Valley in Lebanon. Families were eligible to participate in the study if (1) they had a child aged 9–12 years, and (2) their child scored 17 or more on the intrusion or avoidance scales of the Children’s Revised Impact of Events scale (CRIES-13) [41], a scale validated with children exposed to armed conflict and designed to measure post-traumatic reactions in children. No other exclusion criteria were applied.

### 2.6. Recruitment of Participants and Consent Procedures

Recruitment took place via self-referral. A total of 565 children aged 9–12 years, attending three schools where SSS ran activities, were given a study participant information sheet to take home to their caregivers with an option to ‘opt out’ of the study. No caregivers returned the sheet or showed interest to opt out; therefore, the CRIES-13 [41] measure was administered to all 565 children in the schools by a local research assistant who had been given training in procedures remotely by A.E.-K.

A total of 262 children scored 17 or above on the CRIES-13 measure, indicating clinical levels of post-traumatic stress. Accordingly, 119 of these were randomly selected to be invited to take part in the study along with their families. The remaining 143 children received ‘treatment as usual’, which involved receiving a parenting information booklet to take home to their caregivers, and they were not involved in any further activities. Caregivers of the 119 selected children were sent another information sheet and received a phone call from a research assistant. They were given 3 days to decide and then received another phone call from the research assistant to take their final decision on participation. Five declined the invitation; thus, five further eligible families were invited and all five accepted to take part.

All 119 participating caregivers and their children were invited to attend a baseline measurement session the following week in which written informed consent was obtained and baseline outcome measures were completed in group sessions of 20 families, children, and caregivers in separate rooms.

### 2.7. Randomisation

After completion of the baseline measurements, participants were randomly allocated to the TRT + P group (intervention), the TRT group (active control), or a waitlist control group. In order to ensure allocation concealment, sequentially numbered, opaque, sealed envelopes (SNOSEs) were opened by the local PI after participants agreed to take part in the study and completed a baseline assessment. All data collection was undertaken by researchers blinded to group allocation.

### 2.8. Programme Delivery and Fidelity

The programme was delivered to groups of families by 16 facilitators over five consecutive weeks with parent sessions held every Tuesday and child sessions every Wednesday of the same week. Caregiver sessions were held during school hours, whilst the children’s sessions were held after school hours. A protocol was developed to support the facilitators throughout delivery of the programme, which included the provision of continued supervision and support from the UK via Skype©, Whatsapp©, and email. 

### 2.9. Recruitment of Staff for Intervention and Study Delivery

SSS identified 16 teachers working within their schools who had already been trained on TRT and were available to be trained on the + Parenting component of the enhanced programme. Teachers were trained by the first author (A.E.-K.), an approved TRT trainer and one of the co-authors of the + Parenting materials. Due to sudden unexpected, localised security concerns, planned face-to-face training of the + Parenting intervention was instead conducted via the use of pre-recorded video material and interactive Skype sessions comprising an 8 h split over 2 days. Video material was recorded in the UK by the authors of the + Parenting material and sent online.

### 2.10. Outcomes and Measures

Outcome measures were chosen on the basis of those that were used in previous efficacy and effectiveness trials of TRT and parenting programmes.

#### 2.10.1. Child Mental Health

##### Post-Traumatic Stress

The CRIES-13 [41] is a self-report questionnaire used to screen children at risk for PTSD after traumatic events. The 13-item scale includes three PTSD symptom clusters: intrusion, avoidance, and arousal. Total scores range from 0 to 65 with a cut-off score of 17 or above on the four intrusion and four avoidance items having been found to correctly identify >80% of children with a diagnosis of PTSD [42]. The measure has been used across different cultures and contexts showing good reliability [43].

#### 2.10.2. Depression and Anxiety

The Depression Self-Rating Scale for Children (DSRS) [44] is an 18-item questionnaire measuring depressive symptoms in children. Children are asked to judge whether each statement applied to them over the previous week and then estimate on a three-point scale whether it is true “sometimes”, scored as 1, “never” or “most of the time”, scored 0 or 2 depending on the positive or negative tone of the item. Higher scores indicate a higher level of depressive mood. Good psychometric properties have been demonstrated in child and adolescent populations, including moderate to high internal consistency [45]. 

The Screen for Childhood Anxiety-Related Disorders (SCARED) [46] is a child self-report and caregiver-report instrument to screen children aged 8–18 with anxiety disorders. A total of 41 items reflective of the DSM-IV criteria for anxiety disorders in childhood ask how the child may have felt over the previous 3 months on a three-point scale from “not true or hardly ever true” to “very true or often true”. The scale yields five subscales: panic disorder or significant somatic symptoms, generalised anxiety disorder, separation anxiety, social anxiety disorder, and significant school avoidance. A total score ≥25 may indicate the presence of an anxiety disorder. Both child and parent versions have demonstrated high internal consistency, good test–retest reliability, and satisfactory parent–child agreement [47].

The Strengths and Difficulties Questionnaire (SDQ) [48] is a tool completed by children, caregivers, and teachers to screen children for emotional and behavioural difficulties over the last 6 months. A total of 25 items, rated on a three-point Likert scale ranging from 0 (“not true”) to 2 (“certainly true”), form five subscales, each with five items, including emotional problems, conduct, hyperactivity, peer problems, and prosocial behaviour. Total scores for subscales range from 0 to 5, and the overall total ranges from 0 to 25, with higher scores indicating a higher level of difficulties. Cut-offs of ≥17, 5, 4, 7, and 4 for total difficulties, emotional problems, conduct problems, hyperactivity, and peer problems, respectively, and a cut-off of ≤4 for prosocial behaviour indicate clinical levels of symptoms. Single informant ratings have shown satisfactory reliability and validity [49] across culturally diverse settings [50].

#### 2.10.3. Parenting

The Parenting Scale (PS) [51] is a 30-item measure that assesses parenting skills and confidence. For each item, the parent is presented with a scenario and two different possible ways of managing the situation. The parent is asked to rate on a seven-point Likert scale the behaviour from the two different choices that best describes their style of parenting in response to the scenario during the past 2 months. This measure has three subscales which provide laxness, over-reactivity, verbosity, and total scores. Total scores are calculated by averaging the sum of scores for all items to yield the total score and for subscale items to yield the subscale totals. Low scores indicate high levels of parenting competency, whereas high scores represent dysfunctional parenting styles of caregivers. Satisfactory psychometric properties have been found for this scale, including good reliability and discriminative validity [52].

#### 2.10.4. Caregiver Mental Health

The Impact of Events Scale Revised (IES-R) [53] measures the effects of acute stress and traumas in adults rated on a five-point Likert type scale as 0 (not at all) to 4 (extremely). IES-R is designed to cover three components of PTSD: intrusion, hyperarousal, and avoidance. A total score of 24 or above on this assessment indicates that the respondent has a high likelihood of being diagnosed with full or partial PTSD [54]. Good internal consistency and concurrent and discriminative validity have been found [55].

The Depression–Anxiety–Stress Scale (DASS) [56] is a 21-item self-report measure used to assess the symptoms of depression, anxiety, and stress in individuals. Each item is scored on a scale from 0 (does not apply to me at all) to 3 (applies to me very much). The three subscales (depression, anxiety, and stress) are calculated by generating the sum of their identified item responses. A severity rating scale can be used to ascertain the degree of severity of symptoms of depression, anxiety, and stress, relative to the general population. The validity and reliability of the questionnaire in evaluating the severity of symptoms of depression, anxiety, and stress have previously been proven by several studies [57].

At baseline, a Family Background Questionnaire was used to collect demographic characteristics from caregivers. Measures were completed by caregivers and children at SSS schools immediately after consent was obtained.

At baseline, 5 weeks post baseline (8 weeks post intervention), and 18 weeks post baseline (12 weeks post intervention), all children completed the three outcome measures and caregivers completed the five outcome measures described above. All paper questionnaires and data were entered electronically following data collection and stored securely on SSS premises. Paper questionnaires were transferred to the UK, where data were entered separately by two researchers with support from a bilingual researcher as necessary, and this was crosschecked by a third researcher.

### 2.11. Methods

#### Statistical Analysis

Data were entered and analysed using IBM SPSS for Windows (version 26; IBM, Armonk, NY, USA). Intention to treat (ITT) analysis was used and included all participants as randomised. Data were checked for completeness, and a visual inspection of the histograms, Q–Q plots, and box plots, as well as the calculation of the skewness and kurtosis *z*-values within a range of ±1.96, showed that most data were approximately normally distributed. If not, nonparametric tests were performed. Continuous variables are presented as means and standard deviations, while categorical variables are presented as frequency and percentages. Through Little’s MCAR test, data were proven to be missing completely at random, and data were imputed using the series mean of each variable at each timepoint.

Outcome measures are presented as child-completed and caregiver-completed measures. Child-completed measures comprised CRIES-13, DSRS, and SCARED, while caregiver-completed measures comprised SCARED, SDQ, IES-R, DASS, PS, and PSOC.

For a comparison of means or ranks at the different time points (pre-test, post-test, and follow-up) and among the three groups, i.e., TRT + P, TRT, and waitlist, a two-way repeated-measures ANOVA was used with post hoc Bonferroni corrections. In cases where Mauchly’s test of sphericity indicated that the assumption of sphericity was violated, a Greenhouse–Geisser correction was used. To compare specific mean scores for each outcome measure among three groups, a paired *t*-test at the different timepoints was subsequently used. Friedman’s ANOVA and a Wilcoxon’s signed rank test as a post hoc test were used as nonparametric tests. For comparison of the scores among the three intervention groups at each timepoint, a one-way ANOVA test and the Tukey post hoc test were used. Homogeneity of variances was assured via Levene’s test. A Kruskal–Wallis test was used for nonparametric data comparing all three groups. If significant, a Mann–Whitney U-test was used to compare the three groups individually. To compare categorical data, such as demographic data, a chi-squared test was performed, while Fisher’s exact test was used when the expected frequency was smaller than five in any of the cells. A *p*-value less than 0.05 was taken as significant.

## 3. Results

### 3.1. Characteristics of Study Participants

A total of 119 participants were enrolled in this study; 41 (34.5%) were randomised to the TRT + P group, 38 (31.9%) were randomised to the TRT group, and 40 (33.6%) were randomised to the waitlist control group. The demographic characteristics of study participants are presented in Table 1. Thirty-nine (95.1%) of the caregivers were female and two (4.9%) were male in the TRT + P group, 31 (81.6%) were female and seven (18.4%) were male in the TRT group, and 32 (80%) were female and eight (20%) were male in the waitlist control group.

Regarding marital status, 37 (31.1%) caregivers were married in the TRT + P group and 32 (26.9%) were married in each of the TRT and waitlist control groups. Among all the caregivers, high school was completed by three (2.5%) in the TRT + P group, four (3.4%) in the TRT group, and two (1.7%) in the waitlist control group. Thirty-four caregivers (28.6%) in the TRT + P group, 22 (18.5%) in the TRT group, and 30 (25.2%) in the waitlist control group were not working. All demographics showed no statistically significant differences among the three groups at baseline, as shown in Table 1. Table 2 shows the significant differences at baseline for CRIES 13 Intrusion and the SCARED parent version, with children in the TRT + P having the highest scores.

### 3.2. Feasibility of Engaging and Retaining Families in the Study

**Figure 1:**
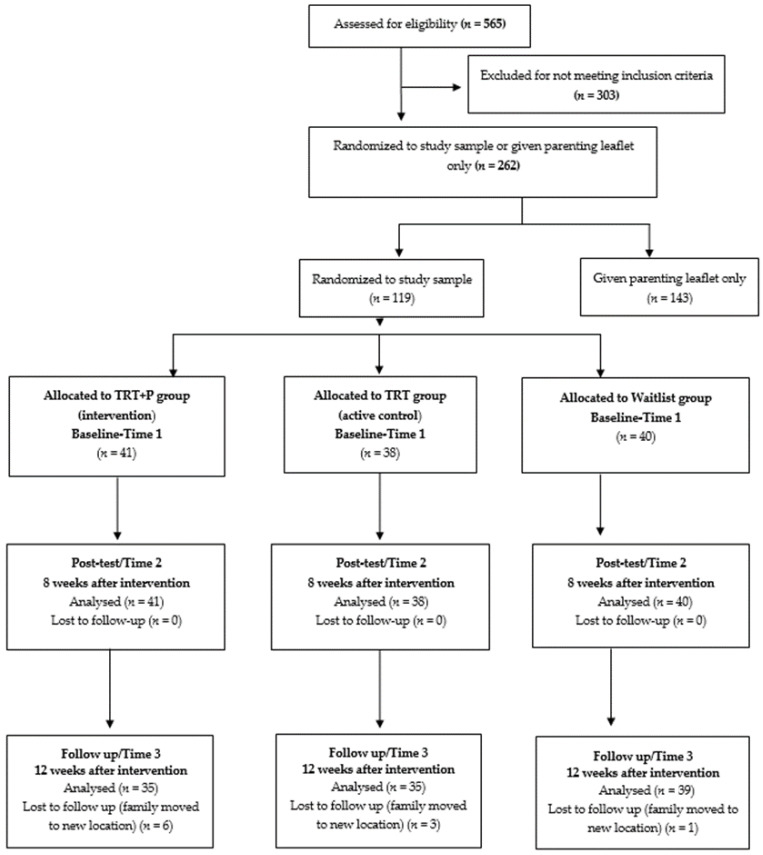
Consort diagram; recruitment of participants, follow-up and missing data over time.

All 119 consented families completed the outcome assessments at both baseline and T2. At T3, assessments were completed by 35 families (85.36%) in the TRT + P group, 35 (92.11%) in the TRT group, and 39 (97.5%) in the waitlist control group (Figure 1).

### 3.3. Child Mental Health

#### 3.3.1. Post-Traumatic Stress

*CRIES-13* **Intrusion**: The TRT + P group had a significantly higher rating on the intrusion score at baseline compared to the TRT (*p* = 0.042) and the waitlist group (*p* = 0.015). Scores were significantly reduced in all three groups at both timepoints after the intervention compared with baseline, with the lowest scores observed at T3, as shown in Table 2. The highest reduction in scores was found in the TRT + P group across all timepoints. TRT + P had significantly lower scores at T2 (*p* = 0.001) and at T3 compared to the waitlist control group (*p* < 0.001). Intrusion scores in the TRT group also reduced significantly compared to the waitlist control group but were only statistically significantly different at T3 (*p* < 0.001). **Avoidance:** As shown in Table 2, baseline scores showed no significant difference at baseline or T2 among the three groups. There was a significant difference at T3 among all three groups, with the highest difference shown between TRT + P and the waitlist control group (*p* < 0.001), followed by the TRT + P group versus the TRT group (*p* = 0.019). The TRT group had significantly lower scores than the waitlist control group at T3 (*p* = 0.049). The overall significant effects were, therefore, mainly attributable to the significant reductions in the TRT + P group. **Arousal:** Scores showed no difference at baseline among the three groups. At T2 and T3, both the TRT + P and the TRT group scores were significantly lower compared to the waitlist control group. Scores dropped highly significantly over all three timepoints in the TRT + P group (t1–t2, t2–t3 and t1–t3; all *p* < 0.001) and in the TRT group (*p*_t1–t2_ = 0.001; p_t1–t3_ < 0.001); however, they remained similar over time in the waitlist control group.

#### 3.3.2. Depression

The Depression Self-Rating Scale for Children (DSRS)

The **total DSRS score** decreased significantly in the TRT + P group (*p*_t1__–__t2_ < 0.001 and *p*_t1__–__t3_ < 0.001) and in the TRT group (*p*_t1__–__t2_ = 0.003 and *p*_t1__–__t3_ = 0.032) but did not change at all in the waitlist control group over time. A significant difference was found at T2 and T3, mainly due to the difference between the TRT + P and the waitlist control group (both times *p* = 0.003).

#### 3.3.3. Anxiety

##### The Screen for Childhood Anxiety Related Disorders (SCARED): Child Version

At baseline, **total SCARED child version scores** were above 34 on average in all three intervention groups, indicating the presence of anxiety disorders (scores ≥ 25). After the intervention, scores reduced significantly in all three groups; however, scores increased again at T3 in the waitlist control group, leading to almost the same levels as those at the start point. Similarly, in the TRT group, scores increased slightly at T3; however, due to the more prominent decrease between T1 and T3, this resulted in an overall significant improvement (*p* < 0.001). Only the TRT + P group decreased in scores over all three timepoints, resulting in the largest improvement overall (*p* < 0.001). Although the three groups did not vary significantly at T1 or T2, both the TRT and the TRT + P group differed significantly from the waitlist group at T3 (*p* = 0.005 and *p* < 0.001). Final scores were well below 25 in the TRT and TRT + P group at T3, but remained above 30 in the waitlist control group.

##### The Screen for Childhood Anxiety Related Disorders (SCARED): Parent Version

The **total SCARED parent version scores** differed significantly among groups at baseline, mainly with the scores in the waitlist control group being significantly lower than in the TRT + P group (*p* = 0.033). These scores reversed over time, with the TRT + P group having the lowest scores at T3 and the waitlist control group having the highest. Hence, from T1 to T3, significant decreases in scores were found in the TRT + P (*p* < 0.001) and TRT groups (*p* < 0.001), compared to an increase in the total score in the waitlist control group (*p* = 0.001). Scores in the waitlist control group at T3 were 33, well above a suggested clinical cut-off of 25, compared to 18.9 in the TRT + P and 22.1 in the TRT group.

#### 3.3.4. Parent-Administered Measures

##### The Strengths and Difficulties Questionnaire (SDQ)

**Total SDQ Difficulty** scores at baseline were in the slightly raised in all three groups and did not differ among the three groups. A significant reduction in scores was found in the TRT + P group between T1 and T2; however, at T3, this decrease was not sustained at a statistically significant level despite scores being lower than at T1. In the other two groups, no significant change was found over time.

### 3.4. Caregiver Mental Health

#### 3.4.1. Post-Traumatic Stress

##### The Impact of Events Scale Revised (IES-R)

The **total IES-R** sum score was 41 at baseline in all three groups, over the clinical cut-off score of 33, which indicates the likely presence of PTSD. All three groups improved significantly between baseline and T2, and between baseline and T3 (*p* < 0.001 for all three groups). At T3, both the TRT and the TRT + P groups improved more than the waitlist control group, with the final scores being at 18.8 and 18.5, compared to 29 in the waitlist control group, being highly statistically significant, as shown in Table 2. The TRT + P group improved faster, whereas the TRT group only caught up between T2 and T3.

#### 3.4.2. Depression, Anxiety, and Stress

##### The Depression–Anxiety–Stress Scale (DASS)

DASS **Stress** levels started off at a moderate level, varying between 21.1 and 24.9 among the three groups (not significant). At T2, no significant changes were found among the three groups; however, at T3, the scores in all three groups declined significantly (*p* < 0.001 in the TRT and TRT + P group and *p* = 0.004 in the waitlist control group), with the lowest scores in the TRT + P group. At T3, the mean score of 12.4 in the TRT + P group differed significantly from the average score in the waitlist control group of 17.6 (*p* = 0.005).

DASS **Anxiety** scores across groups were similar at baseline, ranging between 16 and 17, thus being classified as severe anxiety. The TRT + P group showed a significant decrease in scores (*p* < 0.001) at T2, with a score of 7.1, and, despite a small rise at follow-up, resulted in a highly significant reduction in scores at T3 (*p* < 0.001), bringing the score back to a mild level of anxiety. These effects were also seen in the TRT group, albeit less prominently in scores, reaching the lowest score of 9.9 at T2. At T2, the TRT + P group improved significantly more than the waitlist control group (*p* = 0.005). At T3, both the TRT and the waitlist control groups were still in the category of moderate anxiety, with scores of 10.6 and 12.5.

DASS **Depression**: Participants in the TRT + P and waitlist control groups, but not in the TRT group, were classified as “severely depressed” according to their scores on the depression subscale at baseline. Over time, scores in the TRT + P group indicated the greatest improvements, declining from the highest of all three groups at T1 (24.4) to the lowest scores at T3 (10.5). At T3, the TRT and TRT + P group score was in the “mild depression” category, whereas the waitlist control group participants were still “moderately depressed”.

### 3.5. Parenting Skills and Confidence

#### 3.5.1. The Parenting Scale (PS)

The **total PS** scores were above the clinical cut-off (3.2 points) in all three groups at baseline. Scores in the TRT + P group were significantly reduced from baseline (4.0) to T3 (3.6) (*p* < 0.001). The TRT group scores declined from 3.8 at baseline to 3.6 at T3 (*p* = 0.001), mainly due to the significant reduction between T2 and T3 (*p* = 0.016). The waitlist control group scores did not differ significantly from baseline to T2. At follow-up, the scores of the three groups differed from one another, with the TRT and the TRT + P group scores significantly varying from the waitlist control group (*p* = 0.019 and 0.026).

#### 3.5.2. Parenting Confidence

##### Parenting Sense of Competence (PSOC)

A higher **total PSOC** score indicates a higher parenting sense of competency. At baseline, there was no difference in scores among the three groups. Right after the programme at T2, there was no change in the TRT + P group scores, compared to baseline, whereas the TRT and waitlist control groups worsened significantly (*p* = 0.034 and 0.004). The waitlist control group had the worst scores at T3, being significantly different to the TRT (*p* = 0.002) and the TRT + P groups (*p* = 0.001).

## 4. Discussion

This is the first study to test the combination of the well-established child recovery programme, TRT, with a programme incorporating parenting skills, designed to enhance the capacity of caregivers to support children through extreme life events such as disasters, conflict, and displacement. Furthermore, this study was run with remote training both in the parenting component and in research delivery with Syrian refugees living in the Beqaa Valley in Lebanon.

Recruitment was rapid, and retention throughout the study was high, with both male and female caregivers having participated in the study, an indication of the high affinity and need of families for such a programme. The results showed a highly consistent pattern, with children in the enhanced group showing the greatest levels of improvement, suggesting that the programme design was sound. This was the case for the total scores on all the child measures, including the total difficulty score on the SDQ, and the majority of subscales. For caregivers, this was also the case for the total score on the PS. Significant effects were not found for the PSOC, which measures the parents’ sense of competence. A striking effect, which was not predicted, was the significant effect seen on the parents’ self-report of their own difficulties, with reductions across all areas of the IES-R, showing a significant enhancement with participation in the parenting sessions. Similar effects were seen for DASS depression, anxiety, and stress. Participating in a programme designed primarily to help children cope with their experiences also showed clear benefits for the caregivers’ own mental health. The effectiveness of the enhanced TRT + P intervention is further supported by the fact that assessments by two different raters (caregiver and child measures) both indicated improvements.

These findings demonstrate the value and importance of a brief, carefully designed programme incorporating key components of both trauma recovery and parenting skills. This level of delivery led to significant enhancements in the mental health of both children and caregivers. The findings showed the value of working with the family as a unit, rather than as individuals, supporting an ecologically based view which has been increasingly expressed by many authors [27,28]. The family provides the enduring context in which children grow and develop; enhancing the skills of caregivers to cope with novel challenges and changes in their children’s behaviours and mental health can have an important preventive effect. The enhanced intervention, in addition to improving child mental health, showed the capacity to produce significant improvements in two of the key predictive factors identified by a major review by Scharpf and colleagues [28]: parental mental health and impaired parenting. These findings support research that acknowledges that parents experience psychosocial benefits from parent training interventions even without explicit attention to their own wellbeing [58]. Enhancing the capacity and effectiveness of the family to nurture the child is crucial in low-resource contexts where provision of mental healthcare and support may be extremely limited.

During the second half of each + Parenting caregiver session, caregivers receive a review of and opportunity to practice all the recovery techniques that their children will be undertaking in the child sessions. The primary aim of this is that caregivers are then better able to support their children in practicing the techniques at home. It may be possible that caregivers also benefited from these recovery techniques and this attributed to a significant decrease in caregivers’ self-report of their own difficulties and reductions in depression, anxiety, and stress. Research is underway in the first study exploring the effectiveness of the TRT child sessions with adult refugee populations [59]. As the TRT child sessions are based on the principles of cognitive behavioural therapy and on evidence-based methods for treating trauma, the likelihood of promising results could further support the usefulness of the + Parenting sessions.

The study had its strengths; although it was undertaken with refugees in an area deprived in terms of services and infrastructure, having the presence of a highly skilled NGO such as SSS and the collaboration with the local UNODC office to avail logistical support was essential in ensuring the successful implementation of the trial. In the area selected, there were already considerable numbers of facilitators trained in TRT, as well as a national commitment to support and distribute the programme further. Recruitment and retention for the study were good, and the findings are clear. The nature of the context meant that implementation relied on remote training in the + Parenting component; it is heartening to see the strength of the findings, which would indicate that the novel parenting components can be incorporated successfully and effectively, with brief additional training in these. The significant improvement seen for caregiver mental health shows how the dissemination of recovery techniques can be spread through a family, benefiting multiple people simultaneously.

Running a study in this context involved limitations; it would have been good to have been able to spend time face to face with the research team on the ground, and to monitor the study first-hand. Conditions, resources and security concerns, however, meant that remote training and supervisory processes had to be employed. This also meant that there were limitations on the extent to which process evaluation could be conducted. Some interviews with facilitators were conducted; however, it would have been better to have been able to undertake much fuller and systematic interviews with children and caregivers, as well as facilitators. This may have allowed the highlighting of other domains that the scales potentially were not able to measure. We undertook a measure of the reliability of the scales used across the three timepoints of the assessment. The overwhelming majority (74%) of the Cronbach’s alpha scores generated were around 0.6 (acceptable) and above. Two-thirds of the measures (64%) had Cronbach’s alpha scores over 0.7 (good and above). The only scales that had varying reliability over repeated measures were the scales related to post-traumatic stress in children (CRIES-13) and parenting skills and confidence (PS and PSOC). It is worth noting the increasing reliability score of CRIES over time, where a low Cronbach alpha score was only noted at baseline (Appendix A, Table A1). An in-depth investigation on the reason for this variability will be explored in future studies. Observation of the groups and detailed independent information on fidelity would also have been valuable. Such missed measures of strengthening fidelity may likely have had the potential to strengthen the results of the TRT + P group results, as facilitators were already familiar with TRT, but it was the + Parenting component that was the novel addition. Resources and conditions also constrained the sample size for the study. Even so, with a three-armed trial and a moderate small sample, significant results were found, suggesting powerful effects. The number of children who were screened as eligible and the willingness of caregivers to attend would suggest that a much larger trial is feasible.

### Implications for Utilising Remote Training during and after COVID-19

Prior to the COVID-19 pandemic, there were already insufficient opportunities to reach caregivers in humanitarian contexts with evidence-based family skills programmes [32,60]. This is particularly alarming given the aforementioned significant needs of families in these contexts [25]. During this pandemic, remote teleworking technologies have been utilised to keep all global services working, from business to education and health. There is no doubt that this new exposure will drive a new phase of innovation and global reach of services. This has the potential to be highly beneficial to humanitarian and challenged settings that lacked the accessibility to many such services. However, it is important that the changing service provision platform due to COVID-19 remains able to provide evidence-based programmes with a trained workforce in this new delivery mechanism [61].

Having successfully utilised and established feasibility of the remote training method, this study allows us to be confident in viewing TRT + P as a feasible tool for remote training. For this reason, in September of last year (2020), following the devastating 4 August 2020 blast of the Beirut seaport in Lebanon, and due to COVID-19 travel restrictions, TRT + P training was conducted with new facilitators operating in Beirut via an online platform. Trainer surveys relying on facilitators’ feedback deemed the training successful, and an RCT is currently being undertaken to further explore the effectiveness of TRT + P for children and their caregivers that experienced this disaster [62].

## 5. Conclusions

The study findings indicate that the addition of evidence-based parenting skills components has the potential to enhance the effects of interventions designed to improve children’s mental health in the context of trauma, conflict, and displacement. The parallel improvements in parental mental health carry further public health significance. The results suggest the potential for the use of parenting skills interventions across a wide range of contexts and that remote training is feasible to facilitate quick deployment. The novel + Parenting skills components trialled in this study could be used alongside other interventions addressing parenting under stressful circumstances (including potentially under COVID19 circumstances), with only minimal tailoring.

## Figures and Tables

**Table 1:** Demographic characteristics of study participants at baseline.

Personal Characteristics		TRT + P *N* (%) *n* = 41	TRT*N* (%) *n* = 38	Waitlist *N* (%) *n* = 40	*X* ^2^	*p*-Value
**Gender of Caregiver**	**Male**	2 (1.7%)	7 (5.9%)	8 (6.7%)	4.561	0.102
	**Female**	39 (32.8%)	31 (26.1%)	32 (26.9%)		
**Marital Status of Caregiver**	**Married**	37 (31.1%)	32 (26.9%)	37 (31.1%)	6.694	0.350
	**Divorced/separated**	0 (0%)	1 (0.8%)	0 (0%)		
	**Single**	0 (0%)	2 (1.7%)	0 (0%)		
	**Cohabiting**	0 (0%)	0 (0%)	0 (0%)		
	**Widow**	4 (3.4%)	3 (2.5%)	3 (2.5%)		
**Highest Education**	**Primary school or less**	24 (20.2%)	13 (10.9%)	27 (22.7%)	11.798	0.067
	**Some high school**	13 (10.9%)	15 (12.6%)	8 (6.7%)		
	**Completed high school**	3 (2.5%)	4 (3.4%)	2 (1.7%)		
	**Trade/technical school**	0 (0%)	0 (0%)	0 (0%)		
	**University degree**	1 (0.8%)	6 (5.0%)	3 (2.5%)		
	**Postgraduate degree**	0 (0%)	0 (0%)	0 (0%)		
**Current Employment**	**Not working**	34 (28.6%)	22 (18.5%)	30 (25.2%)	8.695	0.191
	**Full-time job**	1 (0.8%)	6 (5.0%)	5 (4.2%)		
	**Part-time job**	2 (1.7%)	4 (3.4%)	3 (2.5%)		
	**Looking for a job**	4 (3.4%)	6 (5.0%)	2 (1.7%)		
	**Home-based paid work**	0 (0%)	0 (0%)	0 (0%)		
	**N/A**	0 (0%)	0 (0%)	0 (0%)		

**Table 2:** Rating on mental health scales and subscales of children (CRIES 13, DSRS, and SCARED) and caregivers (SCARED, SDQ, IES-R, DASS, PS, and PSOC) by intervention group over time (t1 = pre-test, 1 week before the intervention; t2 = 2 weeks post intervention; t3 = 12 weeks post intervention delivery).

**Child Mental Health**								
**Post-Traumatic Stress**								
**CRIES 13**		***t1***		***t2***		***t3***		***Statistical Test Results***
		***N***	**Mean (SD)**	***N***	**Mean (SD)**	***N***	**Mean (SD)**	
**Intrusion**	TRT + P	41	15.80 (3.41)	41	7.68 (4.03)	35	3.75 (2.53)	F(1.894,219.688) = 174.344 *p* < 0.001 □ ⬟
	TRT	36	13.74 (3.76)	38	8.92 (4.15)	35	5.41 (4.25)	
	Waitlist	40	13.45 (4.08)	40	11.05 (3.92)	38	8.71 (4.09)	
Difference between groups		F(2,116) = 4.729 *p* = 0.011 † ⬟		F(2,116) = 7.197 *p* = 0.001 ⬟		χ^2^(2) = 30.314 *p* < 0.001 □ ⬟		
**Avoidance**	TRT + P	41	14.00 (3.76)	40	8.43 (5.04)	34	4.10 (3.14)	F(2,115) = 77.778 *p* < 0.001 ⬟
	TRT	38	13.32 (3.76)	38	9.74 (5.28)	35	7.19 (5.95)	
	Waitlist	40	12.75 (4.00)	38	10.87 (3.94)	37	8.90 (4.68)	
Difference between groups		F(2,116) = 1.075 *p* = 0.345		F(2,116) = 2.645 *p* = 0.075		χ^2^(2) = 20.931 *p* < 0.001 † □ ⬟		
**Arousal**	TRT + P	41	13.76 (6.63)	40	8.68 (3.90)	34	4.98 (3.54)	F(1.798,208.608) = 42.762 *p* < 0.001 □ ⬟
	TRT	38	12.00 (5.61)	38	7.84 (5.26)	35	6.22 (4.70)	
	Waitlist	40	12.13 (5.68)	39	12.98 (5.76)	39	10.20 (4.66)	
Difference between groups		F(2,116) = 1.071 *p* = 0.346		F(2,116) = 11.894 *p* < 0.001 □ ⬟		χ^2^(2) = 30.085 *p* < 0.001 □ ⬟		
**Depression**								
**DSRS**		***t1***		***t2***		***t3***		***Statistical test results***
		***N***	**Mean (SD)**	***n***	**Mean (SD)**	***n***	**Mean (SD)**	
**Total score**	TRT + P	37	13.62 (5.64)	40	8.35 (5.28)	32	8.73 (4.33)	F(1.806,209.472) = 11.74 *p* < 0.001
	TRT	32	13.32 (5.86)	36	9.67 (4.66)	33	10.57 (5.60)	
	Waitlist	37	11.34 (6.52)	36	11.83 (4.09)	36	12.86 (6.23)	
Difference between groups		F(2,116) = 1.694 *p* = 0.188		F(2,116) = 5.629 *p* = 0.005 ⬟		F(2,116) = 5.586 *p* = 0.005 ⬟		
**Anxiety**								
**SCARED child version**		***t1***		***t2***		***t3***		***Statistical test results***
			**Mean (SD)**		**Mean (SD)**		**Mean (SD)**	
**Total score**	TRT + P		35.14 (12.11)		22.98 (12.17)		20.69 (8.59)	F(2,115) = 34.942 *p* < 0.001
	TRT		34.15 (13.19)		22.56 (14.25)		22.71 (14.75)	
	Waitlist		34.66 (13.69)		27.02 (13.30)		31.99 (14.04)	
Difference between groups		F(2,116) = 0.058 *p* = 0.944		χ^2^(2) = 4.778 *p* = 0.092		F(2,116) = 9.042 *p* < 0.001 □ ⬟		
**SCARED parent version**		***t1***		***t2***		***t3***		***Statistical test results***
		***N***	**Mean (SD)**	***n***	**Mean (SD)**	***n***	**Mean (SD)**	
**Total score**	TRT + P	31	31.52 (13.53)	34	18.51 (11.09)	24	18.93 (7.65)	F(2,115) = 17.412 *p* < 0.001
	TRT	26	29.63 (11.22)	32	23.54 (11.67)	32	22.05 (10.56)	
	Waitlist	25	24.87 (10.37)	35	24.34 (8.32)	34	32.99 (14.73)	
Difference between groups		F(2,116) = 3.396 *p* = 0.037 ⬟		F(2,116) = 3.702 *p* = 0.028 ⬟		F(2,116) = 17.013 *p* < 0.001 □ ⬟		
**Aggression/Behavioural problems**								
**SDQ**		***t1***		***t2***		***t3***		***Statistical test results***
		***N***	**Mean (SD)**	***n***	**Mean (SD)**	***n***	**Mean (SD)**	
**Total Difficulty score**	TRT + P	40	16.75 (5.22)	38	12.75 (4.10)	33	14.17 (5.09)	F(2,115) = 7.389 *p* = 0.001
	TRT	37	15.60 (4.53)	36	14.62 (4.68)	32	13.89 (5.24)	
	Waitlist	37	15.05 (3.63)	37	14.35 (4.36)	37	14.97 (4.13)	
Difference between groups		F(2,116) = 1.485 *p* = 0.231		F(2,116) = 2.132 *p* = 0.123		F(2,116) = 0.526 *p* = 0.592		
**CAREGIVER MENTAL HEALTH**								
**Post-traumatic stress**								
**IES-R**		***t1***		***t2***		***t3***		***Statistical test results***
		***N***	**Mean (SD)**	***n***	**Mean (SD)**	***n***	**Mean (SD)**	
**Total sum**	TRT + P	39	41.27 (16.58)	40	18.39 (8.47)	32	18.54 (8.08)	F(2,115) = 119.064 *p* < 0.001 ⬟
	TRT	32	40.62 (13.62)	37	23.96 (10.12)	35	18.80 (9.89)	
	Waitlist	38	41.46 (12.50)	32	27.12 (13.27)	33	29.00 (14.29)	
Difference between groups		F(2,116) = 0.037 *p* = 0.964		F(2,116) = 6.800 *p* = 0.002 ⬟		F(2,116) = 11.586 *p* < 0.001 □ ⬟		
**Depression, Anxiety and Stress**								
**DASS**		***t1***		***t2***		***t3***		***Statistical test results***
		***n***	**Mean (SD)**	***n***	**Mean (SD)**	***n***	**Mean (SD)**	
**Stress**	TRT + P	41	24.88 (8.31)	41	13.22 (7.92)	34	12.44 (5.77)	F(2,115) = 49.400 *p* < 0.001
	TRT	38	21.16 (9.60)	37	15.59 (9.08)	35	14.27 (6.89)	
	Waitlist	38	23.08 (7.84)	38	17.63 (7.42)	38	17.56 (8.54)	
Difference between groups		F(2,116) = 1.847 *p* = 0.162		F(2,116) = 2.975 *p* = 0.055		F(2,116) = 5.309 *p* = 0.006 ⬟		
**Anxiety**	TRT + P	41	17.46 (12.59)	40	7.05 (8.04)	34	8.48 (4.70)	F(1.814,210.463) = 33.777 *p* < 0.001
	TRT	37	17.00 (10.67)	38	9.89 (9.10)	35	10.62 (8.44)	
	Waitlist	39	16.22 (9.80)	40	10.35 (6.70)	39	12.46 (9.14)	
Difference between groups		F(2,116) = 0.130 *p* = 0.879		χ^2^(2) = 8.350 *p* = 0.015 ⬟		χ^2^(2) = 2.130 *p* = 0.345		
**Depression**	TRT + P	39	24.40 (8.96)	41	10.98 (7.56)	35	10.50 (5.60)	F(1.850,214.655) = 75.631 *p* < 0.001
	TRT	37	19.87 (10.56)	37	12.68 (8.33)	35	11.09 (7.32)	
	Waitlist	37	23.60 (8.37)	40	16.60 (7.95)	38	15.70 (8.82)	
Difference between groups		F(2,116) = 2.623 *p* = 0.077		F(2,116) = 5.306 *p* = 0.006 ⬟		F(2,116) = 5.997 *p* = 0.003 □ ⬟		
**PARENTING**								
**Parent Skills and Confidence**								
**PS**		***t1***		***t2***		***t3***		***Statistical test results***
		***n***	**Mean (SD)**	***n***	**Mean (SD)**	***n***	**Mean (SD)**	
**Total score**	TRT + P	41	4.04 (0.48)	40	3.66 (0.33)	35	3.59 (0.38)	F(1.861,215.909) = 19.972 *p* < 0.001
	TRT	38	3.83 (0.38)	38	3.74 (0.35)	35	3.57 (0.30)	
	Waitlist	40	3.87 (0.39)	40	3.74 (0.33)	39	3.80 (0.38)	
Difference between groups		F(2,116) = 2.762 *p* = 0.067		F(2,116) = 0.858 *p* = 0.427		F(2,116) = 4.825 *p* = 0.010 □ ⬟		
**Parenting confidence**								
**PSOC**		***t1***		***t2***		***t3***		***Statistical test results***
		n	Mean (SD)	n	Mean (SD)	n	Mean (SD)	
**Total score**	TRT + P	36	61.88 (6.56)	39	64.02 (4.73)	32	62.01 (4.50)	F(1.870,216.891) = 3.712 *p* = 0.029
	TRT	36	63.27 (5.36)	36	61.24 (5.61)	30	61.29 (3.00)	
	Waitlist	38	63.16 (5.22)	37	60.20 (3.83)	39	59.88 (6.19)	
Difference between groups		F(2,116) = 0.720 *p* = 0.489		χ^2^(2) = 15.166 *p* = 0.001 † ⬟		χ^2^(2) = 13.413 *p* = 0.001 □ ⬟		

† significant difference between TRT and TRT + P; □ significant difference between TRT and waitlist; ⬟ significant difference between TRT + P and waitlist.

## Data Availability

The data presented in this study are available on request from the corresponding author. The data are not publicly available due to restrictions of privacy.

## Appendix A

**Table A1:** Reliability assessment (Cronbach’s alpha) of scales used over time.

	T1	T2	T3
Child Mental Health			
Post-Traumatic Stress Scale (CRIES 13)			
Intrusion	0.304	0.651	0.766
Avoidance	−0.012 *	0.710	0.818
Arousal	0.557	0.640	0.728
Depression Scale (Total DSRS)	0.782	0.764	0.832
Anxiety Scale (Total SCARED—child version)	0.879	0.928	0.944
Anxiety Scale (Total SCARED—parent version)	0.886	0.895	0.935
Aggression/Behavioural Problem Scale (Total SDQ)	0.550	0.599	0.661
Caregiver Mental Health			
Post-Traumatic Stress Scale (Total IES-R)	0.900	0.902	0.939
Depression, Anxiety and Stress Scale			
Stress	0.793	0.847	0.847
Anxiety	0.862	0.841	0.858
Depression	0.831	0.835	0.837
Parental Skills Confidence Scale (Total PS)	0.408	0.221	0.365
Parental Confidence Scale (Total PSOC)	0.303	0.344	0.422

* Lower reliability linked to one of four items (0.3 when item removed).
